# Quantum Dots: Synthesis, Antibody Conjugation, and HER2-Receptor Targeting for Breast Cancer Therapy

**DOI:** 10.3390/jfb12040075

**Published:** 2021-12-16

**Authors:** Iqra Fatima, Abbas Rahdar, Saman Sargazi, Mahmood Barani, Mohadeseh Hassanisaadi, Vijay Kumar Thakur

**Affiliations:** 1 Department of Pharmacy, Quaid-i-Azam University, Islamabad 45320, Pakistan; iqraf332@gmail.com; 2 Department of Physics, University of Zabol, Zabol 98613-35856, Iran; 3 Cellular and Molecular Research Center, Research Institute of Cellular and Molecular Sciences in Infectious Diseases, Zahedan University of Medical Sciences, Zahedan 98167-43463, Iran; sgz.biomed@gmail.com; 4 Medical Mycology and Bacteriology Research Center, Kerman University of Medical Sciences, Kerman 76169-13555, Iran; mahmoodbarani7@gmail.com (M.B.); mhassanisaadi@gmail.com (M.H.); 5 Department of Plant Protection, Shahid Bahonar University of Kerman, Kerman 76184-11764, Iran; 6 Biorefining and Advanced Materials Research Center, SRUC, Edinburgh EH9 3JG, UK; 7 School of Engineering, University of Petroleum & Energy Studies (UPES), Dehradun 248007, Uttarakhand, India

**Keywords:** breast cancer, HER2 receptor, quantum dots, monoclonal antibodies, quantum dot–antibody conjugate

## Abstract

Breast cancer is becoming one of the main lethal carcinomas in the recent era, and its occurrence rate is increasing day by day. There are different breast cancer biomarkers, and their overexpression takes place in the metastasis of cancer cells. The most prevalent breast cancer biomarker is the human epidermal growth factor receptor2 (HER2). As this biomarker is overexpressed in malignant breast tissues, it has become the main focus in targeted therapies to fight breast cancer. There is a cascade of mechanisms involved in metastasis and cell proliferation in cancer cells. Nanotechnology has become extremely advanced in targeting and imaging cancerous cells. Quantum dots (QDs) are semiconductor NPs, and they are used for bioimaging, biolabeling, and biosensing. They are synthesized by different approaches such as top-down, bottom-up, and synthetic methods. Fully human monoclonal antibodies synthesized using transgenic mice having human immunoglobulin are used to target malignant cells. For the HER2 receptor, herceptin^®^ (trastuzumab) is the most specific antibody (Ab), and it is conjugated with QDs by using different types of coupling mechanisms. This quantum dot monoclonal antibody (QD-mAb) conjugate is localized by injecting it into the blood vessel. After the injection, it goes through a series of steps to reach the intracellular space, and bioimaging of specifically the HER2 receptor occurs, where apoptosis of the cancer cells takes place either by the liberation of Ab or the free radicals.

## 1. Introduction

Cancer is the foremost difficulty of the century, and it has a tremendous impact on the social, mental, and physical lives of human beings [1,2,3]. There is almost a 5% increase in cancer occurrence in developing countries, but this rate is around 1–2% in developed countries [1,4]. Among various types, breast cancer is the most prevalent and the second most deadly type of cancer. Mostly, females of 45 to 55 years of age are affected by it [5,6,7,8,9,10,11]. The transmembrane tyrosine kinase receptor family is termed the human epidermal growth factor receptor (HER family), which is comprised of four types of proteins (HER1–HER4) [12]. In around 20–30% of breast cancer patients, the human epidermal growth factor receptor2 (HER2) receptor is overexpressed. HER2 overexpression leads to a higher relapse rate, more violent disease, and shorter endurance [13,14]. As the overexpression of HER2 occurs in breast cancer, it is the major focus of targeting anticancer therapies [15]. This receptor family is responsible for transmembrane signaling in which cell-cell and cell–stroma communication occurs by signal transduction. Radiotherapy is the major treatment protocol in about 50% of cancer patients [16,17,18,19,20,21].

Nanomedicine is the presentation of technology in the medical field, and lately, it has contributed to the diagnosis and treatment of a variety of diseases (i.e., cancers) [22,23,24,25,26,27,28,29,30,31,32,33]. It is quite possible to deliver the drug to a specific target, which is also known as vectorization. Nanomedicine is used to deliver submicron-sized particles to the target systems for various theranostic purposes [28,34,35,36,37,38,39,40,41,42]. Several nanoparticles (NPs) and nanotechnology-based approaches have been lately introduced for fighting cancer [22,23,27,28,43]. Thanks to unique optical properties, wide excitation spectra, and a very narrow symmetrical intense distribution, it has become possible to apply semiconductor quantum dots (QDs) as a versatile material system with high potential for biomedical applications [44,45,46]. Semiconductor QDs are a novel session of fluorescent constituents. They are used for bioimaging, biolabeling, and biosensing [47]. QDs are more significant than traditional fluorophores. They have higher brightness, fluorescence emission tunability, and less photobleaching. The excitation of multicolored QDs can occur by a single light source and contains broad absorption and narrow emission spectra. The mentioned QDs seem to be the supreme choice for scanning the cell surface receptors. The surface of QDs must be altered by using different biological molecules to make them excellent fluorescent probes [47].

Monoclonal antibodies (mAb) are antigen-specific proteins that bind to a specific ligand [48]. In immunotherapy, monoclonal antibodies (mAb) are bound specifically to antigen cells for cell recognition and selective binding. Monoclonal antibodies have diverse applications in the field of cancer therapy, i.e., for diagnosis and treatment. They are also used in autoimmune diseases for immunosuppression [49]. Trastuzumab is the HER2 receptor-specific antibody (Ab), and after binding to the extracellular domain of HER2, with high specificity, it hinders the proliferation of cancer cells that overexpress HER2 [50]. In QDs antibodies, conjugate antibodies get attached to the surface of inorganic fluorophores through different linkage mechanisms and are used in fluoroimmunoassay [51,52]. Monoclonal antibodies are conjugated with quantum dots by different mechanisms, e.g., either through direct crosslinking or indirect crosslinking. Direct crosslinking occurs when the carboxylic or amino surface groups of QDs are linked to the groups of sulfhydryl or amino on mAbs. In the case of indirect conjugation, there will be the coating of streptavidin on the QDs surface, which gets linked with biotinylated mAbs. Both processes have disadvantages, as the surface functionalization of QDs with unadventurous antibodies by indirect conjugation will help develop giant-sized ligands. These conjugates have inadequate accuracy in detecting the intracellular target. Direct conjugation will result in random mAb orientation and hinder activity. Bioconjugation can also cause denaturation of the Ab molecules [53].

This review summarizes the mechanism of HER2 receptor involvement in breast cancer metastasis. After describing the mechanism of the HER2 receptor, there is a discussion of the preparation methods of QDs and monoclonal antibodies. In the next phase of this review article, there is a brief description of different conjugation approaches used to attach the QDs to the monoclonal antibody (mAb) surface. In the next step, localization of the HER2 receptor by using QDs–monoclonal Ab (QD-mAb) conjugates and the effect produced in response to this targeting has been described. Different in-vitro methods of characterization have also been discussed.

## 2. HER2 Receptor Mechanism

**Figure 1:**
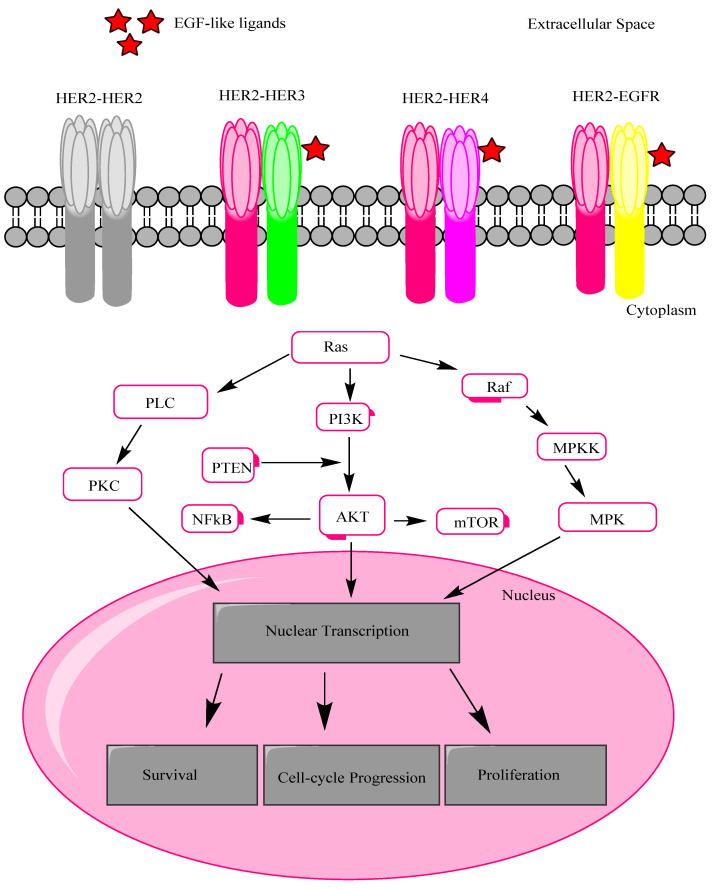
Homo and heterodimerization mechanism. Different transmembrane signals are activated upon homo and heterodimerization, i.e., PI3K/AKT, Raf/MAPK, and PKC pathways, leading to nuclear transcription and modulating the genes associated with cell survivability, proliferation, and cell cycle progression. Here, EGFR, epidermal growth factor receptor; HER, human epidermal growth factor receptor; PLC, phospholipase C; PKC, protein kinase C; PI3K phosphatidylinositol 3- kinase; PTEN, phosphatase and tensin homolog; NFκB, nuclear factor κB; mTOR, mammalian target of rapamycin; MAPK, mitogen-activated protein kinase; MAPKK, MAPK kinase, adapted from ref [54,55].

The monomers of HER receptors go through dimerization and transphosphorylation of their intracellular domains when a ligand binds to their surface. As there is no direct activating ligand of HER2, HER1 and HER3 form heterodimers with it for its activation, or it is activated constitutively. This homo- and the heterodimerization process takes place in the cytoplasm of the cell and leads to the autophosphorylation of tyrosine residue (Figure 1) and results in cell angiogenesis, differentiation, proliferation, survival, and invasion by the activation of signaling pathways, e.g., phosphatidylinositol-4,5-bisphosphate 3 kinases (PI3Ks), mitogen-activated protein kinases (MAPKs), protein kinase C (PKC), etc. [54]. HER2 occurs in an exposed conformation that makes it an excellent dimerization partner for heterodimerization with other family members, and it causes more potent signals than homodimers. When the similar monomers bind together, e.g., HER2–HER2, it is known as homodimerization, and the association of dissimilar monomers such as HER2–HER3, with the help of ligands or other linking agents, is called heterodimerization. The mechanism of conformations of homo- and heterodimers of HER2 is ambiguous, and the way in which these conformations are linked to the dimerization affinities is as of yet undefined. The members of the EGFR group contain a ligand-binding site that allows their mutual linkage. HER3, HER4, and EGFR have a functional ligand-binding site, but HER2 endures a sealed ligand-binding site, and it cannot bind to EGF-like ligands. In the case of homodimerization, as there is no ligand involved, hence the homodimer remains inactive. Interestingly, new crystal structures of Drosophila EGFR (dEGFR) ectoderms are exposed as having asymmetric dimers that contain a single fully shaped ligand-binding site, and the other one is fully or partially closed with one or two ligand-bound subunits. The heterodimers were stable after ligand binding to HER3 or EGFR subunits. After removing the single bound ligand from the HER2 heterodimer, a considerable gap formed in the interface of the dimer.

In the same way, an imitation of the HER2 homodimer indicated the feeble homodimerization of HER2 (Figure 1) [55]. The most potent stimulator of downstream pathways is a HER2–HER3 heterodimer. For example, PI3K/Akt is the chief regulator of cell growth, and endurance is activated by this heterodimer [56]. HER2 can also be activated by interactions with other membrane receptors proteins, such as insulin-like growth factor receptor 1 (IGFR-1) [57].

## 3. Methods of Synthesis of QDs

Different methods are used to prepare QDs, i.e., the top–down method (Table 1), bottom–up method (Table 2), and different synthetic processes. The bulk semiconductor material is used as a precursor. A brief detail of each method is as below.

### 3.1. Using Ultrasonic or Microwave Irradiation

QDs of 1–5 nm size are prepared by passing microwave or ultrasonic radiation from the composition of reactants in water [62]. These aural cavitations create a restricted hotspot by adiabatic compression inside a gas crumpling bubble and lead to the formation of QDs. Zhu et al. formulated 3 nm ZnSe NPs by passing zinc acetate solution in selenourea solution in an argon atmosphere, where the temperature was increased to produce the desired QDs. This process produces QDs with remarkable water-solubility, low toxicity, stability, good reproducibility, and biological compatibility [63].

### 3.2. Hydrothermal Synthesis

Hydrothermal synthesis involves pressure and temperature-controlled crystallization of inorganic salts in water to produce the quantum dots. The QDs prepared by this method give high quantum yield and excellent photostability. This approach is more convenient, efficient, and time-saving. Yang et al. synthesized the high-quality cysteamine-stabilized CdTe QDs using a hydrothermal method [64].

### 3.3. Solvothermal Synthesis

**Figure 2:**
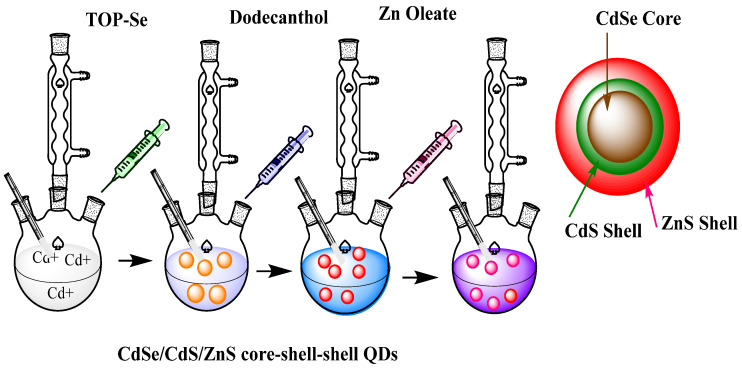
The formation of CdSe/CdS/ZnS core-shell–shell QDs by the solvothermal method. The chemicals used were TOP-Se, dodecanthol, and Zn oleate in a three-necked flask. The specific temperature and vacuum were applied to form these double-shell QDs, adapted from ref [67].

The solvothermal technique is similar to the hydrothermal approach, except that the precursor or reaction solution is not water in this case [65]. Du et al. fabricated CuInSe2 QDs with a diameter below the excitation Bohr radius. The preparation was directed in oleylamine without the presence of organometallic precursors [66]. The formation of CdSe/CdS/ZnS core-shell–shell QDs by the solvothermal method (Figure 2) includes a series of steps. A three-necked flask was weighed down by chemicals such as 0.901 g of oleic acid, 0.103 g of CdO, and 20 mL of tri-n-octylamine. The oxygen and water of the mixture were eliminated by degassing it under vacuum at 150 °C for one hour. Under a N2 environment, the flask was heated to 300°C by the use of a heating mantle, and after that, 0.2 mL of TOP-Se (1 M, Tri-n-octylphosphine base) solution was introduced into the flask at 300 °C. The resulting mixture was shaken for 90 s, and then, 0.1 mL of 1-dodecanethiol and 3 mL of tri-n-octylamine were injected gradually into the flask at the speed of 0.5 mL/min and kept at 300 °C for 40 min. For the formation of the ZnS shell, 8 mL of Zn Oleate solution was introduced above 8 min in the flask with CdSe/CdS core-shell QDs at 100 °C. After that, 0.56 mL of 1-dodecanethiol and 3 mL of tri-n-octylamine were injected dropwise to the flask at the speed of 0.5 mL/minute and kept for 30 min at 100 °C. The solution was examined with the help of ethanol to form CdSe/CdS/ZnS core-shell–shell QDs [67].

### 3.4. Microorganism Biotemplate

The bottom-up approach has been used to synthesize and design particles of a high surface area, having compelling applications in nanotechnology using the biotemplate of microbial origin. Microbial biotemplates have a precise structure and size with high growth and multiplication rates. The combinatorial phage display is used to select the genetically engineered virus that can locate specific surfaces on semiconductors [68].

### 3.5. Electrochemical Assembly

**Figure 3:**
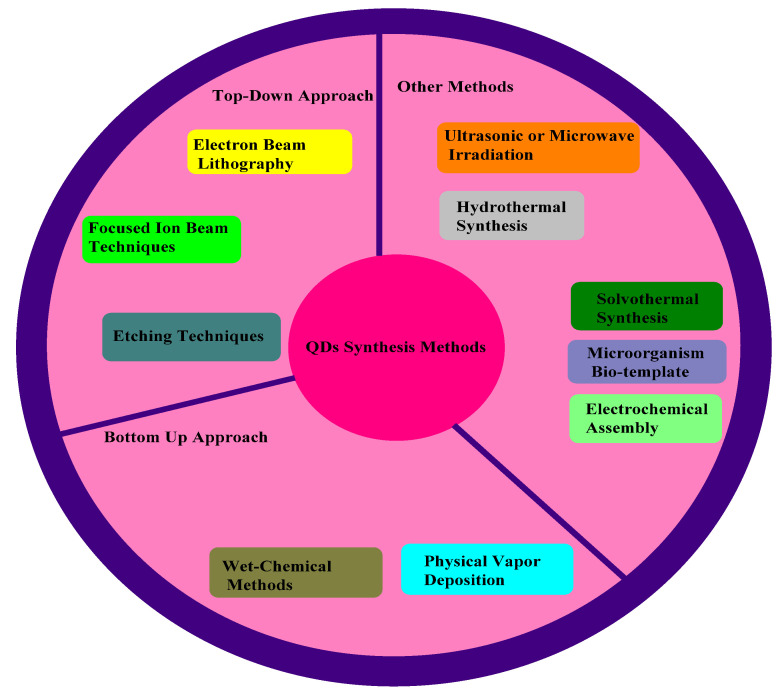
Methods of synthesis of QDs, i.e., top-down approach, bottom-up approach, and other synthetic techniques, adapted from ref [58].

Electrochemical techniques are used to generate self-assembled QDs. A chemical reaction occurs at the junction of electrolyte and metal ions to create a template that leads to the assembly of NPs, i.e., QDs, on the metal surface. Bandyopadhyay et al. studied two electrochemical self-assembly procedures to generate extremely well-ordered quasi-periodic arrays of CdS QDs. The Raman spectra of these self-assembled arrays of Cds QDs were also provided. The QDs prepared by this method show improved defect density, luminescence properties, and crystal quality [69]. Figure 3 shows the synthesis methods of QDs, i.e., the top-down approach, bottom-up approach, and other synthetic techniques.

## 4. Synthesis of Monoclonal Antibodies

**Figure 4:**
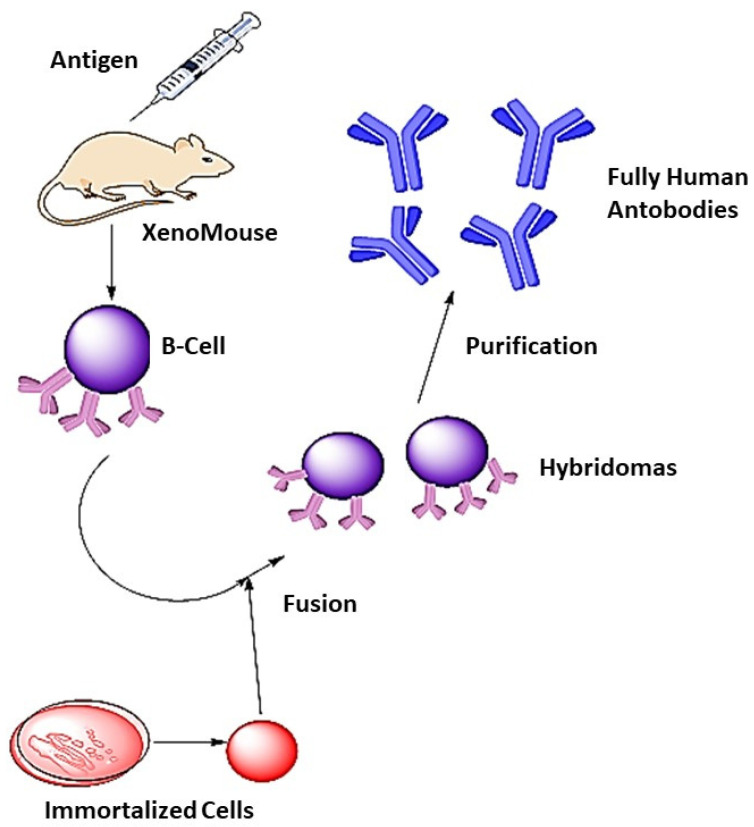
Human antibodies synthesis. A XenoMouse (transgenic mouse capable of producing human antibodies) was injected with antigen. β-cells are isolated and fused with immortalized cells (population of cells from a multicellular organism due to mutation) to produce hybridoma cells which, after purification, yield fully human antibodies, adapted from ref [75].

The traditional hybridoma approach for Ab synthesis is comparatively difficult because it is quite a task to maintain human hybridomas and immortalized cell lines [70]. Now, the screening of Ab libraries is possible by incorporating Ab fragments in filamentous bacteriophages. Similarly, a single fragment (Fab or Fc) is introduced into bacteria to produce monoclonal antibodies [71,72]. Fully human mAb formation acts as a substitute to reengineer murine mAb having a source of low immunogenic antibodies. Phage display technique or transgenic mice are used to form most Ab drugs, but there is no clear difference between them. New human antibodies are prepared mainly by the use of the advanced phage display technique [73]. Human monoclonal antibodies (mAb) can also be prepared by using transgenic mice having human immunoglobulins. The immunization of transgenic mice generates a human Ab response that can form hybridomas to produce human antibodies (Figure 4). The first fully human mAb was Humira^®^, which was intended to treat rheumatoid arthritis [74]. The genetically modified mice and phage display library are the two well-tolerated techniques for the formation of fully human monoclonal antibodies (mAb) [75]. Trastuzumab is a humanized mAb (recombinant) that can attach to the extracellular part of the HER2-tyrosine kinase receptor. In metastatic breast cancer cells, HER2 is overexpressed, which is the best target for trastuzumab. It was approved by Food and Drug Administration (FDA) in 1998 [76,77]. It acts by (i) HER2 receptor internalization and degradation, (ii) Ab-dependent cellular toxicity, and (iii) MAPK and PI3K/Akt interference [78].

## 5. Polymer Coatings and Receptor Localization

QDs used for diagnostic purposes are usually prepared in an organic environment with high temperatures and protected by hydrophobic capping molecules such as trioctylphosphine oxide (TOPO). However, the QDs should be hydrophilic or water-soluble for most biological and bio-analytical purposes. To prepare water-soluble CdSe/ZnS QDs, many different approaches (Figure 3) are used. For example, the use of hydrophilic ligand molecules is common. These ligands include 11-mercaptoundecanoic acid (MUA), polymaleic acid n-hexadecanol ester (PMAH), mercaptoacetic acid (MAA), polymethylmethacrylate (PMMA), mercaptopropionic acid (MPA), dihydrolipoic acid (DHLA), mercaptosuccinic acid (MSA), creatinine, L-arginine, etc. [79,80].

CdSe core QDs coated with ZnS are the most common type of QDs. The core exhibits fluorescence characteristics, and the shell is for the protection and prevention of non-radiative decay and surface flaws. Water-insoluble QDs are converted to hydrophilic dots by applying different types of bifunctional ligand molecules—for example, phosphine, thiol, phosphine, or mono and multidentate ligands—to improve biological properties. On the other hand, QDs can also be coated with hydrophilic or amphiphilic polymers (having both a hydrophilic chain and a hydrophobic side chain) such as polyethylene glycol (PEG). An increased number of functional groups become exposed on the surface, allowing an easy conjugation of different targeting molecules on the surface of QDs [81].

## 6. QDs-Monoclonal Antibody Conjugation Mechanisms

The surface of QDs is coated with specific antibodies to form quantum dot–monoclonal Ab (QD-mAb) conjugates. There are different crosslinking mechanisms for this conjugate formation.

### 6.1. Non-Covalent Linking

Different approaches are used to conjugate QDs with antibodies non-covalently. These methods include adsorption, direct attachment, and the streptavidin-biotin approach.

#### 6.1.1. Adsorption

**Figure 5:**
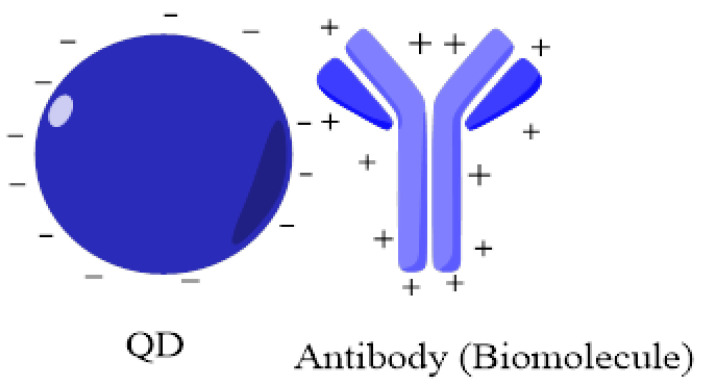
Conjugation through adsorption. Hydrophobic and hydrophilic QDs are attached to the antibodies by different chemical interactions. In the case of hydrophobic QDs, the interaction occurs between amino acids and non-polar molecules, while in the case of hydrophilic QDs, the association occurs by electrostatic interactions adapted from ref [86].

Passive adsorption is a straightforward and traditional approach to bio-conjugate the QDs. Both hydrophilic and hydrophobic QDs can be used in this case. In the case of hydrophobic QDs, adsorption occurs by the association between aromatic amino acids or non-polar molecules with QDs surface coatings. In the same way, the adsorption of hydrophilic particles occurs by the electrostatic interactions between polar molecules and surface coatings of QDs. Proteins undergo a conformational change before attaching with hydrophobic or non-polar particles, as proteins have external hydrophilic molecules [82,83,84]. This is a commonly used approach in which the bond is formed between biomolecules having a positive charge and QDs bearing a negative charge (Figure 5). This process has drawbacks, i.e., weak charge distribution, leading to the detachment and exchange of molecules to another. It is also affected by external factors such as pH, temperature, and ionic strength. Cunha et al. used this method to adsorb negatively charged QDs with lectin to label Candida albicans [83,84,85,86].

#### 6.1.2. Direct Attachment to the Surface

Dative interactions such as thiol interaction and metal-affinity coordination can be used to link the surface of QDs with a biomolecule directly. A coordinate bond shaped by the contribution of electrons of two atoms from a thiol group of biomolecules is called a dative bond. These bonds usually have greater length and less energy than a covalent bond.

Thiol interaction with the QD surface is exhibited by extremely dynamic off-rates, which can restrict conjugation stability over the long run. By raising the number of interactions (i.e., multivalency), these forms of bonds can be strengthened. By motifs, the divalent transition of metals present on the QD surface can be realized to the metal affinity. Unlike covalent interactions, this technique usually results in protein–QD dispersity with no or low particle aggregation. Palomo et al. used this technique to associate QDs with cell glycans [83,85,86].

**Figure 6:**
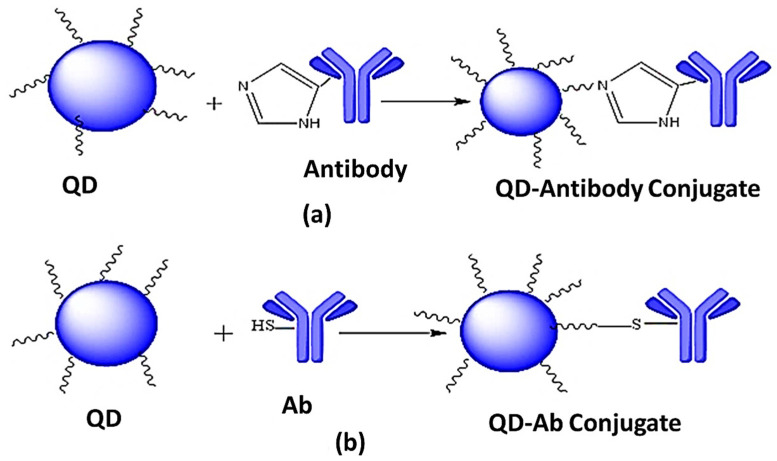
(**a**) Direct attachment to the surface of QDs (QDs are non-covalently conjugated to biomolecules through metal-affinity interactions), (**b**) Direct attachment to the surface of QDs (QDs are non-covalently conjugated through thiol binding) adapted from ref [86].

The imidazole ring at the histidine residue binds with positively charged metal at the QDs surface to form metal-affinity linkages between QDs and biomolecules (Figure 6a). Bio-conjugation can be enhanced by increasing the histidine (His) amino acid of biomolecules. Sulfhydryl groups in thiolated molecules (Figure 6b) can form a dative bond with metal for bio-conjugation. The main disadvantage of this process is the need for thiol residues of biomolecules [87]. Proteins [88,89,90], polymers [91], viruses [91], and carbohydrates [91,92] are the molecules linked through the direct linking process [86].

#### 6.1.3. Streptavidin–Biotin Binding

**Figure 7:**
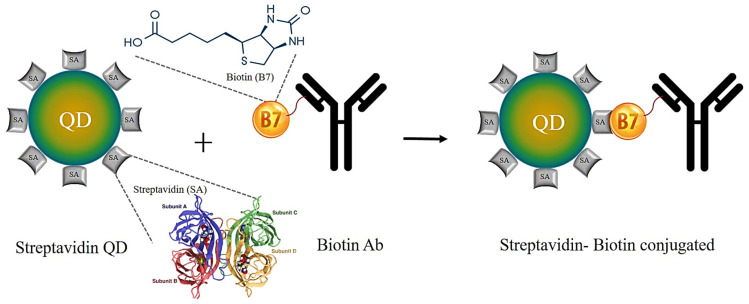
Streptavidin–biotin conjugation. A near-covalent bond is developed between the QDs’ streptavidin and Ab’s biotin, adapted from ref [86].

The high-affinity linkage between streptavidin and biotin has been extensively used for QDs biomolecule conjugation (Figure 7). This method is sometimes mentioned as a covalent bond because it is the strongest non-covalent bond. As this is the near-covalent bond, it is mostly unaffected by pH, temperature, procedure steps, and buffer salts [86,93,94]. Biotin is called vitamin H or B7, and it is a tiny molecule. It is incorporated with biomolecules and does not affect the characteristics of biomolecules. Biotin has a carboxylic group that allows it to be conjugated with different molecules [84]. Streptavidin is a protein having binding potential with biotin. It has four subunits, and each unit has a binding site to biotin. It has less affinity with carbohydrate receptors because it is a non-glycosylated protein. The major disadvantage of this technique is the huge size of the concluding structure.

On the other hand, there is a need to biotinylate the biomolecules for streptavidin-biotin conjugation [84]. There is a need to functionalize the QDs surface with streptavidin so that the biotinylated biomolecule can be attached to it [95]. Antibodies, proteins, peptides, and nucleotides use this approach for bio-conjugation. Ghimire et al. produced QDs-linked polymers through the streptavidin–biotin-binding [96,97].

### 6.2. Covalent Linking

The QDs and antibodies can be covalently linked by three mechanisms, i.e., (i) zero-length coupling, (ii) homobifunctional coupling, and (iii) heterobifunctional coupling.

#### 6.2.1. Zero-Length Coupling

**Figure 8:**
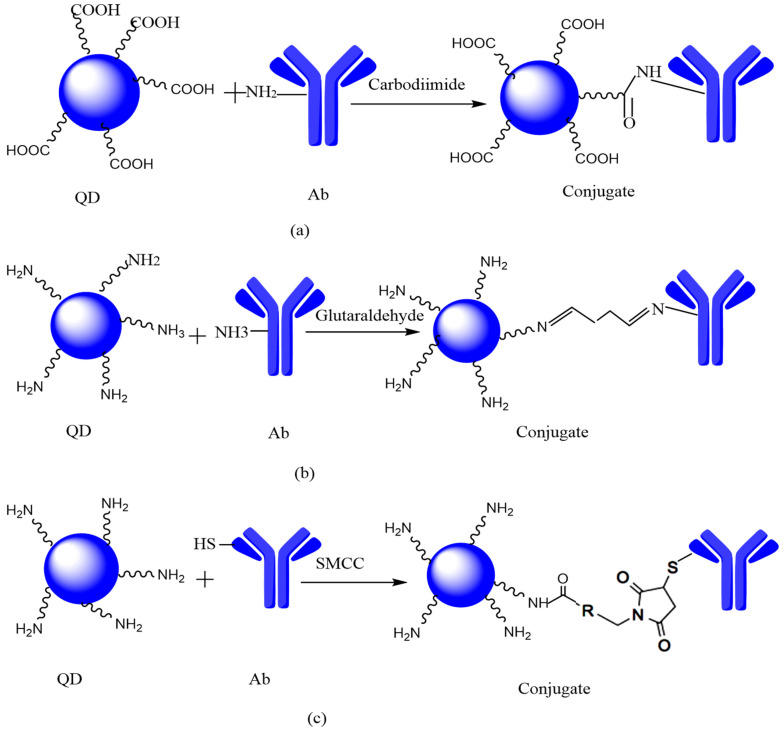
(**a**) Zero-length coupling (QDs are conjugated to biomolecules through a carbodiimide strategy), (**b**) Homobifunctional-coupling (QDs conjugated are to biomolecules using glutaraldehyde), and (**c**) Heterobifunctional coupling adapted from ref [86].

As linking agents in the zero-length coupling, Carbodiimides, by shaping amide bonds, facilitated the reaction of carboxylic acid groups with amine groups. In this case, amino acids are covalently attached through carbodiimide linkage (Figure 8a). Carbodiimides can be either hydrophilic or hydrophobic in nature. The reaction between QDs and antibodies occurs in an aqueous buffer solution, and it requires the use of water-soluble carbodiimide, i.e., EDC (1-ethyl-3-(3-dimethylaminopropyl) carbodiimide hydrochloride). Typically, a preservative is applied beside EDC. The most commonly used preservative or additive with EDC is NHS N-hydroxysuccinimide or sulfo-NHS [82,98]. In this case, the biomolecules get polymerized with many amine and carboxylic groups. This is the major disadvantage of this process. The purification of biomolecules can avoid this polymerization but leads to the denaturation of the biomolecule. To avoid this drawback, the surface of QDs is coated with carboxylic acid and treated with EDC/sulfo NHS [84,94]. Different kinds of biomolecules and NPs use this technique. Yun et al. used EDC/NHS agents to conjugate DNMT1 polyclonal Ab (PcAb) to CdSe/ZnS QDs [86].

#### 6.2.2. Homobifunctional-Coupling

Homobifunctional coupling agents are used to covalently link the two amino groups on the QDs surface and biomolecules. In this case, glutaraldehyde is used to couple biomolecules having amine moieties with aminated QDs. Glutaraldehyde contains two aldehyde groups. These coupling agents have two indistinguishable amine-reactive locations: aldehydes or NHS esters (Figure 8b). This can proceed in either one or two steps. QDs, biomolecules, and coupling agents are mixed simultaneously in a one-step process, so there are chances of amine linkage between two QDs or between QDs or biomolecules. This is the major drawback of this process. In a two-step process, QDs and coupling agents are mixed in the first step; then, there will be a purification step to eliminate the coupling agent. After that, a biomolecule is introduced, and a final bio-conjugate is formed. Zhong et al. used this technique to bind QDs with anti-Escherichia coli antibodies by using glutaraldehyde as a coupling agent. Hydrolysis is the major disadvantage of this process [86]. Different peptides and proteins [99], receptor ligands [100], nucleic acids [101], lectins [102], and antibodies [103] can be conjugated by using this technique.

#### 6.2.3. Heterobifunctional Coupling

A heterobifunctional coupling agent has two reactive groups, one amine and the other sulfhydryl, and they are used to covalently attach a thiol and an amine group (Figure 8c). Activated esters such as NHS ester are used when there is a reaction with an amine. On the other hand, reaction with the sulfhydryl group occurs in the presence of either maleic anhydride or compounds having disulfide bonds [86]. On the thiol-reactive side, mostly maleic existing anhydride derivatives are reacted by the sulfhydryl compounds to form a thioether. The most often used heterobifunctional coupling agent to link biomolecules to the QDs surface is succinimidyl-4-(N-maleimidomethyl) cyclohexane-1-carboxylate (SMCC). This reaction process includes two steps: firstly, the addition of the amine-reactive group (step 1), and then the addition of the thiol-reactive group (step 2). This technique is used to conjugate proteins [104] and antibodies. Brunetti et al. used this technique to link commercial QDs-NH2 with tetra-branched peptides (NT4 peptides) used as cancer therapeutic agents [105].

## 7. HER2 Receptor Targeting by QD-mAB Conjugate

**Figure 9:**
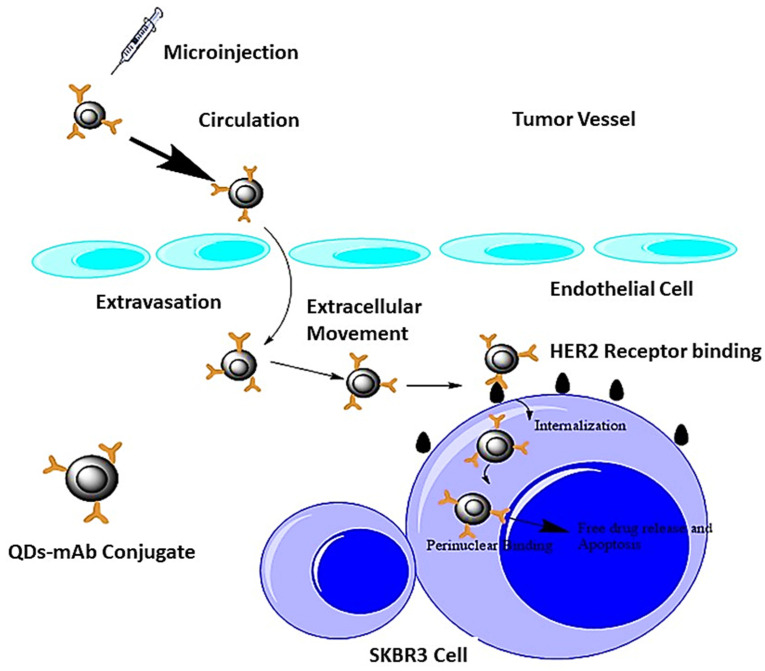
HER2-receptor targeting by QD-mAb conjugate. After injecting into the tumor vessel, the QD-mAb conjugate enters into the endothelial cell through extravasation; then, in a rotating manner, it causes extracellular movements, after which it becomes attached to the HER2 receptor. After that, internalization through vesicle formation occurs, and intracellular binding occurs in the final step, adapted from ref [111].

There are six steps involved in the site-specific transport of QD-mAb conjugate (Figure 9) from tumor vessels to the perinuclear area of the cell. These steps include (i) circulation in the tumor vessel, (ii) extravasation, (iii) drive in the extracellular space, (iv) attach to HER2 receptor located on the cell membrane, (v) endocytosis to move inside of the cell to the perinuclear region, and (vi) the perinuclear region. During the movement through the different sites, the speed of the conjugate was different in different locations. The movement was stop-and-go based, i.e., remaining in the highly restricted region and then moving abruptly. Protein-protein interactions and three-dimensional structures controlled this movement. The motive power generated by blood circulation was used to promote the movement of the conjugate. The conjugate entered into the circulation, and then it was extravagated to the vascular space from interstitial space. After the extracellular movement, the QD-mAb conjugate was attached to the HER2 receptor. After binding to the receptor, it was internalized by endocytosis and reached the perinuclear area. Very little is known about the behavior of NPs inside biological systems. There is a rough estimation of the steps, including blood circulation, cellular localization and movement in the cytoplasm, and reaching the final destination in the target cell or molecule [106,107]. Targeted delivery and uptake feed the cell’s tendency to identify and internalize the QDs conjugated with specific macromolecules (QDs-mAb conjugate) and transport them to intracellular structures such as the nucleus. Advanced techniques such as electroporation enclose the QDs with a nuclear localization sequence and then translocation into the nucleus [108,109]. Antibodies (Trastuzumab in case of HER2) urge the QDs to bind specifically to the HER2 receptor on cancer cell lines (SKBR3 cell lines for HER2 receptor). After binding, QDs leads to programmed cell death and apoptosis by forming reactive oxygen species (ROS) generated by UV or infrared energy emissions. On the other hand, free Ab (Herceptin/Trastuzumab) is also released to cause apoptosis [110]. There are different QDs breast-targeted deliveries discussed in Table 3.

## 8. In-Vitro Characterization of QDS

Photoluminescence spectroscopy is used for the characterization of the optical properties of QDs. Deposited QDs structures were analyzed by using photomodulated reflectance spectroscopy. This approach gives a similar energy resolution profile to that of photoluminescence. The band structure, specifically the wetting layer, can be analyzed by inter-band optical transitions, and a broader range of critical points emphasize the ground state operating at low temperature comparatively [108]. The QDs’ size is the crucial factor determining optical characteristics, and size plays a vital role in regulating the purity and spectral positions of photoluminescence. Dynamic light scattering (DLS), transmission electron microscope (TEM) and transmission electron microscope (TEM) study, and scanning electron microscopic (SEM) analysis are the approaches used to calculate the size of QDs. Scientists have calculated the size and arrangement of CdZnSe/ZnBeSe by using Raman scattering spectroscopies in combination with photoluminescence [117]. Field flow fractionation was also used to characterize hydrophilic QDs [118]. The size of the QDs prepared led to epitaxially different techniques being employed such as TEM analysis, atomic force microscopy (AFM), and scanning tunneling microscopy (STM) [119]. STM was used to calculate the quantum size effect and shapes of InAs and InGaAs QDs [120]. Surface chemistry of QDs is characterized by using Rutherford backscattering, X-ray photoelectron spectroscopy (XPS) study, and nuclear magnetic resonance spectroscopy (NMR) imaging. An extremely robust ultracentrifugation technique was used to characterize the size distribution and surface chemistry of QDs [121].

### 8.1. In-Vitro Characterization of QD-mAb Conjugate

High-resolution transmission electron microscopy (HRTEM) is used to analyze the shape and size of the QDs-mAb conjugate. Highly differentiated cores of QDs can be analyzed after conjugation as large biological molecules are attached to the surface, so there would be enhanced spacing between each QD [122]. Spectrum analysis of QDs with and without the conjugation was analyzed by spectrum analysis to confirm the conjugation process. The appearance of certain peaks at certain wavelengths confirmed the conjugation of QDs surface with antibodies. UV-VIS spectra also show a positive conjugation process as the absorption of the peak lies in the UV range [123,124]. Zeta potential is another analysis that confirms that antibodies occupy the surface. In this case, when a negatively charged ligand binds to the QDs surface, there will be a negative zeta potential. When an Ab binds to the surface, there will be a positive zeta potential value because of the amino groups of the Ab [125,126,127]. Different stretching and bending peaks were analyzed by FTIR analysis to ensure the efficacy of coupling reactions [128]. Morphology, size, binding capability, and molecular weight were determined by atomic force microscopy (AFM), while discontinuous sodium dodecyl sulfate agarose gel electrophoresis (SDS-AGE) was applied to determine molecular biology.

In contrast to the contact mode of AFM, the tapping mode has a less damaging effect on the surface, and it is a very adaptable technique to analyze the surface nanostructure. It is used to study the qualitative and quantitative properties such as the height, angstrom level resolution, shape, and association of surface-adsorbed NPs [129,130]. Here, the heights are measured by topography AFM, while the phase AFM is used to study the morphology of conjugates. Nucleic acid migration, separation of high molecular weight species, and electrophoretic mobility shift assays are performed using AGE [130]. Using this technique and electrophoresis, different biomolecules such as nucleic acids, peptides, and oligonucleotide-modified QDs are analyzed [131]. SDS-AGE molecular weights can elucidate the relative sizes and migration patterns of the QD-mAb conjugate. The size and shape results from AFM can be validated by SDS-AGE [132]. A literature review of HER2 receptor targeting by QDs-mAb conjugates is briefly discussed in the next section.

### 8.2. HER2 Receptor Targeting by QDs-mAb Conjugates

Tada et al. used a single-particle quantum dot linked with cancer-targeting Ab in the tumors of living mice using a dorsal skinfold chamber and a very fast confocal microscope with a highly sensitive camera. QDs conjugated with the monoclonal anti-HER2 Ab were inoculated into the mice having HER2-overexpressing breast cancer to examine the molecular methods of its mechanistic distribution to the tumor. By using a dorsal skinfold chamber, the drive of single complexes of the QDs-Ab could be evidently detected at 30 frames per second inside the tumor. Six procedures of delivery were examined efficaciously such as (i) firstly in the blood vessel or circulation region, (ii) extravasation, (iii) in the extracellular environment, (iv) on the cell membrane for binding to HER2, (v) movement from the cell membrane to the perinuclear section, and (vi) in the perinuclear region. The photo analysis of the movement processes of single particles in-vivo gives evidence on therapeutic nanoparticles conjugated to the Ab, which can enhance therapeutic efficacy [111]. Seung-Jin et al. immobilized a classic monoclonal Ab, Herceptin, on the surface of CdSe/ZnS core-shell QDs to increase their particular connections with breast cancer cells (SK-BR3). Dynamic light scattering was used to detect the average size of the core-shell QDs, i.e., 28 nm. This size showed an increase up to 86 nm after the immobilization of Herceptin. The in-vitro cell culture testing indicated that the keratin-forming cancer cells (KB) multiplied significantly in the existence of Herceptin-linked QDs (QDs-Her, 5 nmol/mL).

In the same way, most of the breast cancer cells (SK-BR3) were found dead. The interface of QDs-Her with SK-BR3 cells was examined, by confocal laser scanning microscopy, to elucidate the cell death. The QDs-Her was linked particularly to the membrane of SK-BR3 cells, and the membrane became saturated after 6 h of incubation. This indicates that the specific binding of Herceptin to the HER2 receptor of the SK-BR3 membrane completely inhibited the growth signal of breast cancer and resulted in cell death [110]. Takashi-Jin et al. used QDs conjugated antibodies that are the most significant fluorescent probes. A simple process was used to conjugate the Ab on the surface of QDs. ProteinA was applied as an adaptor protein to bind the Ab to the QDs for surface imaging. Numerous types of antibodies can easily bind to the surface of QDs by non-covalent attachment between the Fc (fragment crystallization) part of the Ab after their linkage with ProteinA. HER2 in KPL-4 human breast cancer cells was marked by the use of anti-HER2 Ab-linked ProteinA-QDs to indicate the usefulness of ProteinA-conjugated QDs. The multiplexed imaging of CXCR4 (chemokine receptor) and HER2 in KPL-4 cells was carried out. The result indicated that CXCR4 receptors co-occur with HER2 receptors in the surface membrane of KPL-4 cells. In living cells, the multiplexed imaging of surface receptors is performed by ProteinA-facilitated Ab conjugation to QDs [47].

Tatsiana et al. performed immunolabeling of breast and lung cancer cell lines by ultra-small and shiny nanoprobes associated with QDs conjugated to single-domain anti-HER2 antibodies. Their efficiency was compared to traditional organic dyes (Alexa Fluor 568 and Alexa Fluor 488)-labeled anti-HER2 monoclonal antibodies. The single-domain antibodies conjugated to QDs (sdAbs-QDs) attained greater staining in a board of lung cancer cell lines having distinct HER2 expression. This indicates their excellent perspective for the progress of more sensitive assays for the initial examination of cancer biomarkers [133]. Rizvi et al. characterized the near-infrared-emitting QDs, and their in-vitro toxicity was proven by using three cancer cell lines such as SK-BR3 (HER2-overexpressing), MCF7 (HER2-underexpressing), and HepG2. Mouse anti-human anti-HER2 monoclonal Ab was linked to the near-infrared-emitting QDs. In-vitro toxicity testing indicated the biocompatibility of MCF7 and SK-BR3 cell lines at 60 µg/mL concentration after one hour and 24 h of exposure using near-infrared-emitting QDs. HER2 receptors were effectively localized on SK-BR3 cells by near-infrared-emitting QDs anti-HER2 Ab bioconjugates [128].

## 9. Applications of QDs

**Figure 10:**
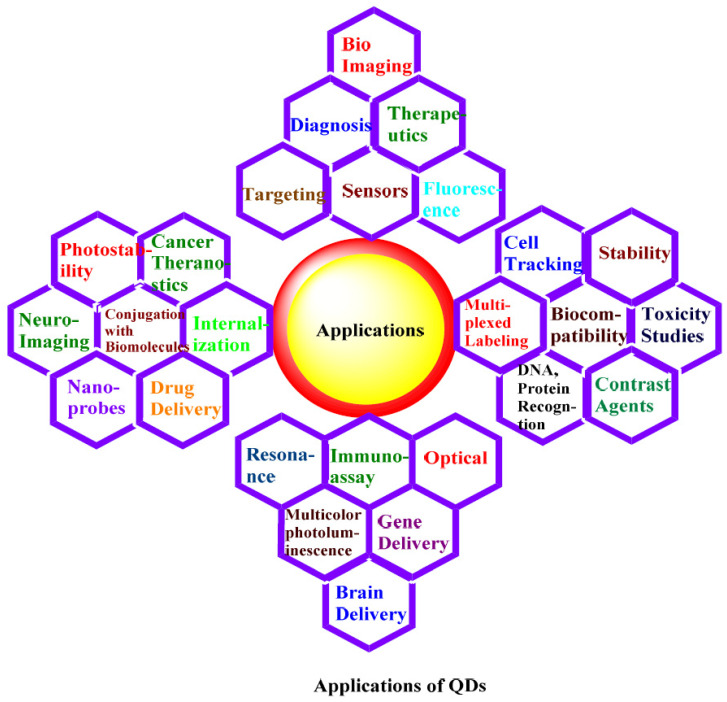
Applications of QDs, adapted from ref [142,143].

QDs have multiple biodiagnostic, bioimaging, photodynamic therapy, and targeted delivery applications. Some of the applications of QDs are summarized in Figure 10 [134,135]. Specific receptors are targeted by QDs coated with receptor-specific moieties such as folate, which was conjugated to the surface of QDs for receptor-specific targeting [136]. The QD–biomolecule (e.g., folate) conjugates were noticed in mouse lymphoma after some time of incubation. A precise targeting was observed by folate at folate receptors demonstrated by the increase in fluorescence concentration in non-specific QDs. There will be an increased expression of folate receptors in cancer cells, and this range is determined by folate, a nutrient. Then, this degree of folate expression will be used to diagnose cancer, as the overexpression is a sign of malignancy [137].

Photostable QDs are used to record images for extended periods compared to fluorescent dyes because they have a lower photobleaching effect. Cd containing QDs was visible even after several days after intraocular injection using in-vivo imaging approaches [138]. For the in-vivo experiment, mice were injected with these QDs subcutaneously, and fluorescence was determined in brain areas, specifically. In this case, the highest fluorescence was noticed after three days, and it lasted for seven days. The exact position of the conjugates was determined at subcellular level resolution. It was observed that the QDs were internalized into various cell types, as their internalization of QDs is considered a novel technique in in-vivo neuroimaging studies [137]. Live cell imaging and detection can be performed by QDs as well.

Both external and internal imaging of cells is possible. Different techniques such as cell-mediated delivery and surface modifications are used to determine cell imaging and internalization applications [139,140]. The QDs can be tracked into human breast cancer cells [111]. Anti-HER2 antibodies conjugated with QDs were used to locate the NPs in tumor cell blood vessels in mice. The whole process, including the conjugate movement in blood vessels, then extravasation step, movement in the extracellular cellular environment, adhesion to HER2 receptors on the cell surface, and internalization step, was visualized. This process is a novel technique and can track different biomolecules and comprehend subcellular movements [137]. QDs are used to track cancer cells in-vivo in metastasis [141]. QDs coated with tumor-targeting antibodies have been developed and were injected into mice. The in-vivo studies showed the accumulation of these conjugates into the tumoral cells. Meanwhile, in another approach, the QDs-labeled tumoral cells were injected into mice, and in both cases, they were tracked by multiphoton microscopy. In both cases, the results indicated the multicolor in-vivo visualization [142].

Multiplexed cell labeling is another use of QDs. After conjugation of QDs with antibodies such as anti-HER2, EGFR, and mTOR antibodies, the cancer cell lines were nurtured with QD-Ab conjugates for one hour by Yezhelyev et al. Cell lines were marked with five different kinds of QDs, and it was found that the cancer biomarkers were present either on the cell membrane or in the nucleus of the cell. Further breast cancer cell analysis confirmed that by computing the fluorescence intensity of QDs, the extent of quantitative appearance of cancer biomarkers could be calculated concurrently [144]. Förster resonance energy transfer (FRET) is a very influential technique used to study biological interactions that works through QDs [145]. There should be simplistic conjugation approaches used, and coatings must be water-soluble to conjugate the biological molecules with QDs. Stable and biocompatible QDs are produced to be incorporated in this technique. Immunohistochemistry (IHC) has been used to diagnose and predict human cancer for the last 80 years [146]. In this case, multicolored molecular profiling is not possible. Multiplexing is essential to define the cancer stage, as it can show more than one type of biomarker simultaneously. IHC is a semi-quantitative technique leading to inter-observer variations in results. QDs have played a unique role in multiplexed fluorescent labeling. It is a very informal and convenient technique. Multiple colored QDs are excited simultaneously, and their emission is used as an advanced photostable approach for imaging the samples [146,147]. Exclusive physical and optical characteristics make QDs extraordinary candidates for biosensing and bioimaging, as they can attach many biological molecules on their surface. QDs are used in modern DNA and protein recognition methods; in recent years, a QD marked with aptamer (a nucleic acid) was used to unmark adenosine triphosphate (ATP). Three carcinoma cell lines, HepG2, SK-BR-3 (HER2 overexpressing), and MCF7, were used to characterize near-infrared (NIR)-emitting QDs and determine their in-vitro toxicity (HER2 underexpressing). A mouse’s anti-human anti-HER2 mAb was coupled to QDs that generate near-infrared (NIR) light [128]. The safety of NIR-emitting QDs at a 60 g/mL concentration was confirmed by exposing cancer cells for one h and 24 h under in-vitro toxicity experiments. QD–antiHER2–Ab bioconjugates that produce NIR light successfully targeted HER2 receptors on the surface of carcinoma cells (SK-BR-3 and MCF7 cell lines) [128]. Another study used QD immunohistochemistry (QD-IHC) to measure ki67. This study exhibited the significance of QD-IHC to predict breast cancer by detecting HER2-positive (non-luminal). The identification of breast cancer was conducted by looking at the expression of the Ki67 marker in 108 breast cancer tissues (non-luminal) with HER2-positive by using both QD-IHC and IHC. QDs-IHC to a found antigen location acts as traditional IHC. Both techniques have a reasonable correlation of staining rates (r = 0.993). Additionally, these methods found an excellent agreement of measurements (=0.874) of Ki67 expression (cutoff: 30%) in breast cancer. However, for the Ki67 score, the QD-IHC method demonstrated more high-grade inter-observer agreement than traditional IHC. Ki67 expression levels above 30% were linked to shorter disease duration (in a disease-free state), particularly in lymph node-negative. Ki67 detection and assessment were made more accessible and more accurate using QD-IHC imaging. The Ki67 score was shown to be an independent predictor of survival in breast patients with HER2-positive (non-luminal) [148]. Different advantages and disadvantages of QDs usage in breast cancer-targeted deliveries are summarized in Table 4.

## 10. Conclusions and Outlook

This review summarizes the targeting of the cancer biomarker HER2 receptor by using quantum dot monoclonal Ab conjugate (QD-mAb). QDs can be used for nucleic acid detection, drug treatments, real-time imaging of cancer cells, and a wide range of other biomedical applications. QDs are considered the most advanced technique for cell labeling and imaging. Different preparation methods of QDs have been discussed, e.g., the top-down approach and the bottom-up approach.

The mAb preparation from XenoMouse was performed, as the conventional techniques are time consuming. Trastuzumab is the main Ab used for HER2-receptor targeting. There are different coupling techniques to conjugate the surface of QDs with biomolecules. The conjugation of Ab to QD helps selectively locate the tumor cells and destroy them either by free radicals or free Ab release. HER2 receptor localization, which is the article’s main point, is elaborated as the migration of the conjugate from the blood vessel to the intracellular space. In-vitro characterization techniques of the QDs and QD-mAb conjugates and some in-vivo studies and applications of QD Ab conjugate have been deliberated.

As a result of their outstanding optical properties, the use of QDs in breast research will increase shortly. QDs-based imaging techniques have demonstrated their promising applicability in the simultaneous quantification of breast cancer biomarkers. These biomarkers, such as HER2 receptors, are upregulated in breast tumors and can be detected using these innovative methods even in the tumor microenvironment.

Numerous reports have already shown the successful use of QDs against breast cancer; however, concerning their in-vivo application, caution must be taken due to the undesirable toxicity of QDs’ components. Still, the efficient delivery of these multifunctional biomaterials and their conjugates is profoundly affected by their nature and the type of the studied cells. Therefore, developing relevant safety regulations is needed for commercialization. There are some obstacles in using QDs for breast cancer therapy, such as their relatively large size for medical imaging, the stereospecific blockade effect, in-vivo aggregation of QDs, and their toxic effects. Still, we firmly believe that these nanostructures are more beneficial to nanomedicine to dismiss, and biocompatible QDs will ultimately be available for routine clinical use in breast cancer patients.

## Figures and Tables

**Table 1:** The synthesis of ODs using electron beam lithography and focused ion beam (FIB) techniques, as well as an etching technique such as the top–down approaches.

Top-Down Approach				
Method	Procedure	Advantages	Particle Size (nm)	Ref
Electron beam lithography	Beam of electron strikes with electron-sensitive film	A high degree of flexibility	≈30	[58]
Focused ion beam (FIB) techniques	FIB functioned at high or low beam currents for imaging and can be operated at site-specific sputtering	Ultra-small radius particles	8–20	[58,59]
Etching techniques	Following the trapping of reactive gas species in an etching system, the formed plasma breaks down the gas molecules into more reactive fragments with the assistance of a controlled radiofrequency voltage	Close-packed arrays formed	40	[58,60]

**Table 2:** The synthesis of QDs using the physical vapor (PVD) approach and the wet-chemical method as two bottom-up approaches.

Bottom–Up Approach		
Method	Description	Ref
Wet-chemical methods	Precipitation technique using one or more solvents. Different process parameters are carefully controlled, and QDs of specific size and shape are prepared by a controlling ratio of cationic and anionic species, temperature, and double-layer thickness. Bhand et al. synthesized CdTe QDs using the wet-chemical method and employed characterization techniques to investigate their optical, compositional, and morphological properties. The resulting QDs were in size range of 2.5–4 nm from the absorption peak position.	[61]
Physical vapor deposition (PVD)	Condensation is the phenomenon involved in this case. The most commonly used PVD technique is sputtering. In PVD, a solid or liquid source is used to vaporize the material and transported to a vacuum, and then condensation occurs. Melvin et al. synthesized CdSe/CdTe colloidal QDs by a physical vapor deposition method. The PVD method has previously been used to generate niobium oxide (Nb_2_O_5_), CdSe/CdTeQDs, and other nanocrystals.	[58]

**Table 3:** QDs-based breast cancer targeted (HER2 receptor-targeted) deliveries.

Targeted Deliveries	Details	Ref
Near-IR QDs-loaded micelles	The high absorption of the near-IR QDs-loaded micelles by HER2 receptor-overexpressing carcinoma cells, which are distributed fast in-vivo.	[112]
Inorganic nanoparticles with a core/shell configuration. Metals (e.g., iron oxide, gold, and QDs) or organic fluorescent dyes encapsulated in silica form the core, while metals or organic polymers form the shell	The HER2 receptor is exploited to target magnetic NPs in breast cancer tissue for imaging and diagnostic purposes.	[113]
Biomarker-targeted fluorescent probes	Breast cancer cells and associated animal models are scanned using fluorescent probes. Fluorescent probes are superior to other diagnostic methods in terms of high selectivity, sensitivity, specificity, signal intensity, and early diagnosis.	[114]
QDS-based inorganic NPs for HER2 receptor targeting	The application of targeted multimodal NPs as vectors in therapeutic purposes, sources of signals in diagnostic purposes, and monitor endogenous response simultaneously.	[115]
QDs in breast cancer therapy	As a result of their ultra-sensitivity, bio-conjugated QDs were used to detect metastasis, especially micro-metastasis. QDs may also be employed to track circulating tumor cells or even circulating stem cells, which are essential prognosis factors and sources of cancer recurrence.	[116]

**Table 4:** Advantages and disadvantages of quantum dots (QDs) usage in breast cancer-targeted deliveries.

Advantages	Disadvantages	Ref
Biological imaging, diagnosis, and therapeutic applications	High toxicity	[149]
High photostability and no photobleaching	In high breast cancer cell lines, QDs are epigenetic and genotoxic carcinogens	[149]
Versatility for bio-conjugation with various biomolecules	Detection or diagnosis of cancer is limited to superficial sites	[150]
In comparison with organic fluorophores, the QDs amplify signal brightness up to 10–100 times more	As a result of the oxidative breakdown of its heavy metal core, metal ions will be released into the environment, where they will attach to the thiol functional group of intracellular proteins and cause the function of subcellular organelles to be impaired	[150]

## Data Availability

Data are included within this article.
